# Supplementary material for: Self-tests for COVID-19: What is the evidence? A living systematic review and meta-analysis (2020–2023)
Source: PLOS Glob Public Health. 2024 Feb 7;4(2):e0002336. doi: 10.1371/journal.pgph.0002336 (PMC10849237; doi:10.1371/journal.pgph.0002336)
Supplement: S1 Box — (DOCX) [file pgph.0002336.s001.docx]

**S1 Box Search String**

Pubmed:

("covid 19*"[All Fields] OR "covid*"[All Fields] OR "sars cov 2*"[All Fields]) AND ("self test*"[All Fields] OR "self test*"[All Fields] OR "self screen*"[All Fields] OR "self screen*"[All Fields] OR "home test*"[All Fields] OR "at home test*"[All Fields] OR "at home test*"[All Fields])

Embase Current:

((COVID-19* or covid* or "SARS-CoV-2*") and ("Self-test*" or "Self test*" or "Self-screen*" or "Self screen*" or "home test*" or "at home test*" or "at-home test*")).mp. [mp=title, abstract, heading word, drug trade name, original title, device manufacturer, drug manufacturer, device trade name, keyword heading word, floating subheading word, candidate term word]

Web of Science Core Collection:

((COVID-19* OR covid* OR "SARS-CoV-2*")) AND (("Self-test*" OR "Self test*" OR "Self-screen*" OR "Self screen*" OR "home test*" OR "at home test*" OR "at-home test*")) (All Fields)
