# Supplementary material for: Self-tests for COVID-19: What is the evidence? A living systematic review and meta-analysis (2020–2023)
Source: PLOS Glob Public Health. 2024 Feb 7;4(2):e0002336. doi: 10.1371/journal.pgph.0002336 (PMC10849237; doi:10.1371/journal.pgph.0002336)
Supplement: S1 Table — (DOCX) [file pgph.0002336.s002.docx]

**S1 Table Outcomes Description**

| **Outcomes** | **Definition** |
| --- | --- |
| Sensitivity | The ability of COVID-19 self-tests to accurately diagnose the infection in individuals compared to conventional testing. |
| Specificity | The ability of COVID-19 self-tests to accurately identify people with absence of infection compared to conventional testing. |
| DOR | The odds of having a COVID-19 positive self-test in individuals with the infection relative to individuals without the infection. |
| Feasibility | The participant’s convenience and completion of COVID-19 self-testing (self- sampling, test conduction, interpretation, reporting, and linkage to care) measured by completion rate, usability index, and ease of the testing process. |
| Acceptability | The proportion of participants who chose or were willing to choose self-testing when offered the option. |
| Impact outcomes | A change in outcomes following a self-test result such as TAT and linkage to care. |
| Preference | The proportion of participants who preferred self-testing over conventional testing options. |
