# Supplementary material for: Self-tests for COVID-19: What is the evidence? A living systematic review and meta-analysis (2020–2023)
Source: PLOS Glob Public Health. 2024 Feb 7;4(2):e0002336. doi: 10.1371/journal.pgph.0002336 (PMC10849237; doi:10.1371/journal.pgph.0002336)
Supplement: S2 Table — (DOCX) [file pgph.0002336.s003.docx]

**S2 Table Study Characteristics**

| **Study ID** | **First Author** | **Year** | **Country of Study** | **Study Design** | **Sample Size** | **Type of Screening/ Testing Effort** | **Study Setting** | **Main Findings** |
| --- | --- | --- | --- | --- | --- | --- | --- | --- |
| 1 | Harmon, Alexander | 2021 | The United States of America (USA) | Cohort | 257 | Targeted Screening (school, college, university, nursing home, sports club etc.) | At-home | Sensitivity within days 0 to 12 of symptom onset = 78.9% (95%CI: 69.1-88.8%), Specificity = 97.1% (95%CI: 96.3-97.8%); Twice-weekly serial self-testing detected 15 infections with 96.2% sensitivity for day 0-3 of symptom onset; Allowed activities in co-working laboratory sites to continue safely |
| 2 | Lindner, Andreas | 2021 | Germany | Cohort, prospective study of diagnostic accuracy | 146 | Mass screening (targeted at a broader community, general population) | Supervised (observed, but no intervention) | Sensitivity = 82.5%; Sensitivity increased to 96.6% at high viral loads; COVIDST was easy to perform |
| 3 | Mistler, Colleen | 2022 | USA | Cross-sectional | 110 | Targeted Screening (school, college, university, nursing home, sports club etc.) | Not applicable | High willingness and acceptability |
| 4 | Betsch, Cornelia | 2021 | Germany | Cross-sectional | 4,026 | Mass screening (targeted at a broader community, general population) | At-home | Difficulties in correctly interpreting test results; Incentives can increase willingness to COVIDST |
| 5 | Bien-Gund, Cedric | 2021 | USA | Cross-sectional | 586 | Targeted Screening (school, college, university, nursing home, sports club etc.); secondary distribution of COVID-19 self-testing kits,1,2 whereby at-risk or infected individuals distribute test kits to contacts in their social network | Not applicable | High motivation to distribute and use COVIDST kits |
| 6 | Bouillat, Caroline | 2021 | France and Cameroon | Cross-sectional | 283 | Screening at a healthcare facility | Not applicable | Delivery of self-tests was acquired in pharmacies despite initial reluctance; training required to distribute and use these tests in a relevant and appropriate manner |
| 7* | Thomas, Caroline | 2022 | Indonesia | Qualitative | 53 | Targeted Screening (school, college, university, nursing home, sports club etc.) | Not applicable | COVIDST is a welcome screening tool; regulated sales and counterfeit kits checks needed |
| 8 | Thomas, Caroline | 2022 | Indonesia | Cross-sectional | 630 | Mass screening (targeted at a broader community, general population) | Not applicable | COVIDST is acceptable; can complement an over-burdened healthcare system |
| 9 | Hughes, David | 2022 | The United Kingdom (UK) | Cohort | 7310 | Targeted Screening (school, college, university, nursing home, sports club etc.) | Supervised, At-home | Inequalities in testing; if prevalence is low, confirmatory testing is needed |
| 10 | Mouliou, Dimitra | 2021 | Greece | Cross-sectional | 614 | Mass screening (targeted at a broader community, general population) | Not applicable | Concerns regarding COVIDST such as reliability, cost, effectiveness; COVIDST should be used along with symptom assessment, physician diagnosis, and confirmatory laboratory testing |
| 11 | Schuit, Ewoud | 2021 | The Netherlands | Cross-sectional, diagnostic accuracy study | 2,950 | Mass screening (targeted at a broader community, general population) | At-home | Hangzhou AllTest AgRDT was not reliable while SD Biosensor demonstrated high sensitivity in symptomatic individuals and in those without a prior SARS-CoV-2 infection; Low sensitivity of nasal COVIDST in asymptomatic individuals and in those previously infected with SARS-CoV-2 |
| 12 | Lamb, Georgia | 2021 | UK | Cohort | 5076 | Targeted Screening (school, college, university, nursing home, sports club etc.) | At-home | Innova LDF had high PPV amongst hospital staff when COVID-19 prevalence was high; allows earlier isolation of infected workers and facilitates early detection who are asymptomatic/have other symptoms |
| 13 | Phillips, Gregory | 2021 | USA | Cross-sectional | 932 | Targeted Screening (school, college, university, nursing home, sports club etc.), for sexual and gender minorities | Not applicable | Critical disparities observed for sexual and gender minority (SGM) populations in COVID-19 symptomology, at-home testing, and prevention interest |
| 14 | Moller, Ida Johanne | 2022 | Denmark | prospective diagnostic accuracy study | 827 | Mass screening (targeted at a broader community, general population) | At-home | COVIDST reported to be reliable, user-acceptable, and safe to use by laypeople; can be used as a supplement to professional testing |
| 15 | Hirst, Jennifer | 2021 | UK | Cohort | 734 | Targeted Screening (school, college, university, nursing home, sports club etc.) | Unsupervised, At-home | Repeat COVIDST was acceptable, feasible, and allowed accurate self-test interpretation |
| 16 | Frediani, Jennifer | 2021 | USA | Cross-sectional, diagnostic accuracy study | 309 | Mass screening (targeted at a broader community, general population) | Supervised, Unsupervised | BinaxNOW can accurately detect new COVID-19 variants; user-errors may lower COVIDST sensitivities |
| 17 | Stohr, Joep | 2021 | The Netherlands | Cross-sectional, diagnostic accuracy | 3201 | Mass screening (targeted at a broader community, general population) | At-home | COVIDST with BD-RDT and Roche-RDT have a high specificity and a relatively high sensitivity to identify highly contagious individuals |
| 18 | Wachinger, Jonas | 2021 | Germany | Qualitative | 130 | Targeted Screening (school, college, university, nursing home, sports club etc.) | Unsupervised, At-home | At-home COVIDST is feasible and acceptable; can supplement conventional testing to facilitate a safe return and face-to-face teaching at schools |
| 19 | Wanat, Marta | 2021 | UK | Cohort, Qualitative | 734 | Targeted Screening (school, college, university, nursing home, sports club etc.) | Supervised, Unsupervised, At-home | Clear messaging was effective in highlighting the benefits of regular COVIDST and in identifying asymptomatic cases; Concerns need to be addressed regarding safety, convenience of testing, ability to do tests for successful scale-up |
| 20 | Cassuto, Nino Guy | 2021 | France | Cross-sectional | 335 | Screening at a healthcare facility | Supervised | COVID-VIRO is a relevant tool for COVIDST in the general population with a high usability, ease of use, and excellent diagnostic performance |
| 21 | Goggolidou, Paraskevi | 2021 | Greece | Cross-sectional, Qualitative | 248 | Unknown | Not applicable | High acceptability; saliva testing is preferable; Concerns such as accessible reporting, contact tracing infrastructures, central registration, and validation of COVIDST kits for implementation required for effective scale-up |
| 22 | Kheiroddin, Parastoo | 2021 | Germany | Qualitative | 16,808 | Targeted Screening (school, college, university, nursing home, sports club etc.) | Unknown | COVIDST was not as effective repeated gargle pool rRCT-PCR testing in schools |
| 23 | Willeit, Peter | 2021 | Austria | Cross-sectional, DAS | 784707 | Targeted Screening (school, college, university, nursing home, sports club etc.) | Supervised | COVIDST can detect a subset of infected individuals with low-moderate sensitivity; high specificity was reported for non-infected negative participants |
| 24 | Denford, Sarah | 2021 | UK | Qualitative | 52 | Targeted Screening (school, college, university, nursing home, sports club etc.) | At-home | High motivations to test with COVIDST kits; Acceptability affected by factors such as needing to avoid self-isolation, concerns with low sensitivity, perceived benefits; Self-testers reported positive consequences following COVIDST, ability to engage in essential activities, uncertainty, self-isolation while testing |
| 25 | Hoehl, Sebastian | 2021 | Germany | Cross-sectional | 711 | Targeted Screening (school, college, university, nursing home, sports club etc.) | Unsupervised, At-home | COVIDST by schoolteachers allowed early SARS-CoV-2 detection; 76.2 false positives detected; COVIDST was effective in symptomatic populations with high COVID-19 prevalence |
| 26 | Kim, S | 2021 | Republic of Korea | Cross-sectional, Diagnostic Accuracy Study | 296 | Targeted Screening (school, college, university, nursing home, sports club etc.) | Unsupervised, At-home | High sensitivity, especially for symptomatic populations reported for COVIDST; Sensitivity decreases as CT value increases; screening most effective in early phase of disease onset |
| 27 | Tonen-Wolyec, Serge | 2021 | France | Cross-sectional | 106 | Screening at a healthcare facility | Supervised | 90.9% sensitivity and 100% specificity; high agreement, reliability, accuracy, usability for supervised COVIDST |
| 28 | Woloshin, Steven | 2022 | USA | Randomized Controlled Trial | 338 | Mass screening (targeted at a broader community, general population), general population but limited sample size | Not applicable | At-home self-testers may not follow government advisories post testing, producing unintended risks and unnecessary disruptions; scientific and evidence-based instructions needed. |
| 29 | Prazuck, Thierry | 2021 | France | Cross-sectional, Diagnostic Accuracy Study | 119 | Screening at a healthcare facility | Supervised | Self-tests were comfortable and easy to use; less trauma; faster results |
| 30* | Zwart, V.F. | 2022 | The Netherlands | Cohort | 7196 | Targeted Screening (school, college, university, nursing home, sports club etc.) | Unsupervised | Sensitivities were 61.5%, 50.3%, and 74.2% for different study groups; Higher sensitivity with combined oropharyngeal - mid-turbinate sampling; Reliability varied with the sampling method and the presence of COVID-19 symptoms |
| 31 | Undelikwo, Veronica A | 2022 | Nigeria | Qualitative | 58 | Targeted Screening (school, college, university, nursing home, sports club etc.) | Not applicable | Self-testing may assist in the prompt detection of cases; perceived inefficiency of the health systems may limit self-testers' access to psychosocial and clinical support. |
| 32 | Love, Nicola | 2021 | UK | Cross-sectional, Qualitative | 812 | Mass screening (targeted at a broader community, general population) | Unsupervised, At-home | High acceptability, compliance, and positivity rate of self-testing |
| 33 | Martin, Alex F | 2021 | UK | Cross-sectional, Qualitative | 524 | Mass screening (targeted at a broader community, general population) | Unsupervised, At-home | Self-testing is feasible, may facilitate contact testing, and can promote self-isolation adherence |
| 34* | Peto, Tim | 2021 | UK | Cross-sectional | 372 | Mass screening (targeted at a broader community, general population) | Unknown | Innova LFD has good sensitivity with excellent specificity; barriers include kit failure rates and lack of pre-test training |
| 35 | Downs, Louise | 2021 | UK | Cross-sectional | 8657 | Screening at a healthcare facility | Unsupervised, At-home | Positive predictive value of self-tests was 96% and false positive rate was 0.03%; less sensitive than PCR but detected cases with higher viral loads; reduced staff-to-staff and staff-to-patient transmissions |
| 36 | Tulloch, John | 2021 | UK | Cross-sectional | 1638 | Targeted Screening (school, college, university, nursing home, sports club etc.) | Supervised | Poor adherence to self-testing protocol due to test integration and procedural factors, socio-economic factors, cognitive overload and the emotional value of testing |
| 37 | Institute of Population Health, University of Liverpool | 2021 | UK | Cohort | 387,580 | Mass screening (targeted at a broader community, general population) | Unsupervised, At-home | Daily self-testing saved 8,292 key worker workdays; Self-test uptake was lowest among young children (5-9 years) and older people (70+, particularly 80+ years), in deprived areas, in students, and areas with fewer digital resources or lower digital literacy. |
| 38 | Sibanda, Euphemia | 2022 | Zimbabwe & Malawi | Cross-sectional, Qualitative, mixed-methods | 1,204 | Mass screening (targeted at a broader community, general population) | Unsupervised | High usability and acceptability of self-testing in general and health-care worker populations in low- and middle-income countries; Pre-test demonstrations were useful |
| 39 | García-Fiñana, Marta | 2021 | UK | Cohort | 5869 | Mass screening (targeted at a broader community, general population) | Supervised | Asymptomatic self-testing had a sensitivity of 40.0% specificity of 99.9%; Clear and accurate information on self-test interpretation needed |
| 40 | Shilton, Sonjelle | 2022 | Brazil, India, Indonesia, Kenya, Nigeria, Peru, Philippines and South Africa | Qualitative | 9403 | Mass screening (targeted at a broader community, general population) | Not applicable | High willingness and support for use of COVIDST although this may vary by country |
| 41 | Jairoun, Ammar Abdulrahman | 2022 | UAE | Cross-sectional | 876 | Mass screening (targeted at a broader community, general population) | Not applicable | High acceptability and willingness to use OTC vending machines to access self-test kits; Barriers to COVIDST include potential costs and ease of access, especially among people who can't read or understand instructions |
| 42 | Soni, Apurv | 2022 | USA | Cohort | 5674 | Mass screening (targeted at a broader community, general population), Serial Testing | Unsupervised, At-home | COVIDST performance is not inferior in participants infected with Omicron variant as compared to Delta variant; Serial testing improved sensitivity |
| 43 | Rader, Benjamin | 2022 | USA | Cross-sectional | 418,279 | Mass screening (targeted at a broader community, general population) | Unsupervised, At-home | COVIDST along with other preventive measures can reduce spread of infection; Access to COVIDST can be improved by providing reliable and low-cost or free at-home test kits to underserved populations |
| 44 | Herbert, Carly | 2022 | USA | Cohort | 206 | Targeted Screening (school, college, university, nursing home, sports club etc.) | Unsupervised, At-home | High adherence to COVIDST, significant reliability, high acceptability and feasibility of smartphone app assisted self-testing |
| 45 | Herbert, Carly | 2022 | USA | Cohort | 313000 | Mass screening (targeted at a broader community, general population) | Unsupervised, At-home | Reporting of COVIDST results can be improved by digital application-based reporting with incentives |
| 46 | Herbert, Carly | 2022 | USA | Cohort | 3496 | Mass screening (targeted at a broader community, general population) | Unsupervised, At-home | High COVIDST adherence and self-test result reporting |
| 47 | LeRouge, Cynthia | 2022 | USA | Cross-sectional | 260 | Targeted Screening (school, college, university, nursing home, sports club etc.) | Not applicable | Facilitators to smartphone supported COVIDST included its quality, personal capacity to perform self-testing, and feelings of empowerment and activation |
| 48 | D'Agostino, Emily | 2022 | USA | Cross-sectional | 2591 | Mass screening (targeted at a broader community, general population) | Not applicable | Motivators to COVIDST included testing before gathering, close contact with an infected person, positive household member, and unvaccinated status; Disparities existed by race and other socio-economic factors which could be reduced by increasing COVIDST test access and uptake |
| 49 | Schuit, Ewoud | 2022 | The Netherlands | Cross-sectional | 6497 | Screening at a healthcare facility | Unsupervised, At-home | COVIDST sensitivities decreased for omicron variant; Performance increased after addition of oropharyngeal to nasal self-sampling |
| 50 | Marinos, Georgios | 2022 | Greece | Cross-sectional | 1000 | Targeted Screening (school, college, university, nursing home, sports club etc.) | Unsupervised, At-home | High adherence to serial COVIDST among students with 70% needing assistance; high compliance although older age adolescents were less likely to comply |
| 51 | Martinez-Perez, Guillermo | 2022 | Brazil | Cross-sectional | 417 | Mass screening (targeted at a broader community, general population) | Not applicable | Mass testing with COVIDST was favorable, acceptable; Majority of self-testers were likely to report self-test results, request facility-based post-test counseling, self-isolate, and warn their close contacts |
| 52 | Papenburg, Jesse | 2022 | Canada | Cross-sectional | 647 | Targeted Screening (school, college, university, nursing home, sports club etc.) | Supervised | COVIDST accuracy was poor when manufacturer instructions were used; modified instructions was associated with better test performance |
| 53 | Ritchey, Matthew | 2022 | USA | Cross-sectional | NA | Mass screening (targeted at a broader community, general population) | Unsupervised, At-home | COVIDST is a valuable risk-reduction tool that can guide individual actions but currently has limited utility in enhancing public health surveillance |
| 54 | Leventopoulos, Michail | 2022 | Greece | Cross-sectional, diagnostic accuracy study | 833 | Screening at a healthcare facility | Supervised | COVIDST sensitivity, specificity and accuracy rates were, 98.18%, 100.00%, and 99.28%, respectively |
| 55 | Coker, M. O. | 2022 | USA | Cross-sectional | 475 | Targeted Screening (school, college, university, nursing home, sports club etc.) | Not applicable | COVIDST of dental professionals, coworkers, and patients were perceived to provide safety at 49%, 55%, and 68%, respectively. |
| 56 | Coller, Ryan J. | 2022 | USA | Cohort | 102 | Targeted Screening (school, college, university, nursing home, sports club etc.), screening for children with medical complexities | Supervised, At-home | High feasibility of COVIDST in a pediatric cohort with neurologic impairment and chronic respiratory failure |
| 57 | Qasmieh, Saba | 2022 | USA | Cross-sectional | 1,030 | Mass screening (targeted at a broader community, general population) | At-home | Self-test positivity was 5.2%; can contribute to hidden prevalence of cases |
| 58* | Nwaozuru, Ucheoma | 2022 | USA | Cross-sectional, Qualitative | 28 | Mass screening (targeted at a broader community, general population) | Not applicable | COVIDST was acceptable; barriers include accuracy and costs of tests |
| 59 | Agusti, Christina | 2022 | Spain | Cross-sectional | 492 | Targeted Screening (school, college, university, nursing home, sports club etc.) | Unsupervised, At-home | Implementing online COVID-19 self-testing in schools and healthcare settings in Spain is feasible. |
| 60 | Bae, Seongman | 2022 | South Korea | Longitudinal DAS, cohort or cross-sectional? | 34 | Targeted Screening (school, college, university, nursing home, sports club etc.) | Unknown | Half of COVID-positive participants gave negative RAT results. However, the remaining cases were detected by RAT; high negative predictive value for viable viral shedding was reported |
| 61 | Dallera, Giulia | 2022 | UK | Cross-sectional | 1093 | Targeted Screening (school, college, university, nursing home, sports club etc.) | Unknown | Professionally witnessed home-based videoed testing was highly acceptable and feasible; COVIDST informed participants' decision making and supported the safe reopening of live mass events at full capacity. |
| 62 | Daniore, Paola | 2022 | Switzerland | Cross-sectional | NA | Mass screening (targeted at a broader community, general population) | Unsupervised, At-home | Self-testing app combined with digital contact tracing impacted participants' motivations to self-test with COVIDST and worked reasonably well |
| 63 | Fishman, Jessica | 2022 | USA | Cross-sectional | 4299 | Unknown | Not applicable | Political beliefs affected motivations to self-test wherein right and center politics were more likely to prefer self-testing than those with left politics preference |
| 64 | Hajek, Andre | 2023 | Germany | Cross-sectional | 3075 | Mass screening (targeted at a broader community, general population) | Not applicable | COVIDST was not perceived as uncomfortable; Facilitators included protection of others, own health precautions, and traveling; Barriers included a lack of perceived benefit; high satisfaction observed |
| 65 | O'Byrne, Patrick | 2022 | Canada | Cohort | 3653 | Targeted Screening (school, college, university, nursing home, sports club etc.) | Unsupervised, At-home | Delivering COVID self-tests via HIV self-test kits distribution website provided tests to BIPOC communities and highlighted the utility of such systems for delivering testing in specific cohorts during future pandemics. |
| 66 | Qasmieh, Saba A | 2022 | USA | Cross-sectional | 4328 | Mass screening (targeted at a broader community, general population) | Unsupervised, At-home | Cases were missed by government surveillance systems during the Omicron B.1.1.529 surge, when at-home testing was common; the self-test reporting system needs to be enhanced to capture all cases |
| 67 | Schilling, Josh | 2023 | USA | Case Control | 822 | Mass screening (targeted at a broader community, general population) | Unsupervised, At-home | AI-enabled COVID-19 self-testing tool was highly acceptability, usable and preferred across diverse socioeconomic populations |
| 68 | Stemler, Jannik | 2022 | Germany | Cross-sectional | 419 | Mass screening (targeted at a broader community, general population) | Unsupervised, At-home | Reported prevalence of COVID-19 infections was reported to be higher as a result of unreported self-test results or only PCR results being reported in the government statistics |
| 69 | Venekamp, Roderick P | 2022 | The Netherlands | DAS, Cross-sectional | 3600 | Screening at a healthcare facility | Unsupervised, At-home | COVIDST sensitivity in asymptomatic individuals in the Omicron period was very low. |
| 70 | Wu, Fan | 2023 | China | Cross-sectional | 5107 | Mass screening (targeted at a broader community, general population) | Not applicable | Moderate intention to use COVIDST and acceptance was found with significant differences in diverse sociodemographic populations; barriers include reliability, testing method, price and authority |

* Indicates studies which are not peer-reviewed and are available as pre-prints.
