# Supplementary material for: Self-tests for COVID-19: What is the evidence? A living systematic review and meta-analysis (2020–2023)
Source: PLOS Glob Public Health. 2024 Feb 7;4(2):e0002336. doi: 10.1371/journal.pgph.0002336 (PMC10849237; doi:10.1371/journal.pgph.0002336)
Supplement: S3 Table — (DOCX) [file pgph.0002336.s004.docx]

**S3 Table Diagnostic accuracy across test devices**

| **Study ID** | **Author** | **Study population** | **Symptoms** | **Index test** | **Reference Standard** | **Sample Size** | **Sensitivity** | **Specificity** |
| --- | --- | --- | --- | --- | --- | --- | --- | --- |
| 1 | Harmon 2021 | Laboratory staff | Symptomatic | E25 Bio Inc | RT-PCR | 2951 | 78.57 | 97.06 |
| 2 | Lindner et al. 2022 | General population | Symptomatic | Standard Q (SD Biosensor) | RT-PCR | 144 | 81.71 | 99.52 |
| 11 | Schuit et al. 2021 | General population | Symptomatic | SD Biosensor (Roche) | RT-PCR | 2306 | 78.33 | 99.52 |
| 11 | Schuit et al. 2021 | General population | Symptomatic | Hangzhou Alltest Biotech | RT-PCR | 484 | 50.99 | 98.82 |
| 11 | Schuit et al. 2021 | General population | Asymptomatic | Hangzhou Alltest Biotech | RT-PCR | 2319 | 26.56 | 99.67 |
| 11 | Schuit et al. 2021 | General population | Asymptomatic | SD Biosensor (Roche) | RT-PCR | 487 | 23.44 | 99.45 |
| 14 | Moller et al. 2021 | General population | Symptomatic | DNA Diagnostic | RT-PCR | 72 | 75.00 | 99.09 |
| 14 | Moller et al. 2021 | General population | Symptomatic | Hangzhou Immuno Biotech | RT-PCR | 329 | 67.65 | 97.83 |
| 14 | Moller et al. 2021 | General population | Asymptomatic | Hangzhou Immuno Biotech | RT-PCR | 79 | 44.12 | 99.84 |
| 14 | Moller et al. 2021 | General population | Asymptomatic | DNA Diagnostic | RT-PCR | 343 | 40.91 | 99.85 |
| 16 | Frediani et al. 2021 | General population | Symptomatic | Binax Now | RT-PCR | 44 | 55.88 | 98.28 |
| 17 | Stohr et al. 2021 | General population | Both | Roche-RDT | RT-PCR | 1556 | 61.40 | 99.68 |
| 17 | Stohr et al. 2021 | General population | Both | BD-RDT | RT-PCR | 1583 | 49.15 | 99.82 |
| 26 | Kim et al. 2021 | Hospital patients | Both | Standard Q (SD Biosensor) | RT-PCR | 296 | 94.38 | 99.77 |
| 27 | Tonen-Wolyec et al. 2021 | General population | Asymptomatic | Biosynex | RT-PCR | 30 | 97.92 | 91.67 |
| 27 | Tonen-Wolyec et al. 2021 | General population | Symptomatic | Biosynex | RT-PCR | 76 | 90.63 | 96.88 |
| 30 | Zwart et al. 2022 | Healthcare workers | Both | Standard Q (SD Biosensor) | RT-PCR | 5216 | 81.99 | 99.35 |
| 30 | Zwart et al. 2022 | Healthcare workers | Both | BD-RDT | RT-PCR | 2258 | 63.66 | 96.95 |
| 34 | Peto 2021 | Trained Operators | Unknown | Innova | RT-PCR | 7747 | 66.31 | 99.68 |
| 39 | Garcia-Finana et al. 2021 | General population | Asymptomatic | Innova | RT-PCR | 5504 | 40.14 | 99.94 |
| 49 | Schuit et al. 2022 | General population | Symptomatic | Flowflex (Acon Labs ) | RT-PCR | 620 | 78.97 | 97.16 |
| 49 | Schuit et al. 2022 | General population | Symptomatic | MPBio (MP Biomedicals) | RT-PCR | 820 | 69.87 | 98.82 |
| 49 | Schuit et al. 2022 | General population | Symptomatic | CLINITEST (Siemens Healthineers) | RT-PCR | 726 | 70.16 | 99.33 |
| 49 | Schuit et al. 2022* | General population | Symptomatic | MPBio (MP Biomedicals) | RT-PCR | 544 | 83.01 | 97.77 |
| 49 | Schuit et al. 2022** | General population | Symptomatic | CLINITEST (Siemens Healthineers) | RT-PCR | 653 | 77.25 | 96.97 |
| 54 | Leventopoulos et al. 2022 | General population | Both | Rapid SARS–CoV–2 Antigen Test Card, home test (Boson) | RT-PCR | 833 | 98.18 | 100.00 |
| 69 | Venekamp et al. 2022 | General population | Asymptomatic | Flowflex (Acon Labs ) | RT-PCR | 1229 | 27.46 | 99.81 |
| 69 | Venekamp et al. 2022 | General population | Asymptomatic | MPBio (MP Biomedicals) | RT-PCR | 1027 | 20.87 | 99.78 |
| 69 | Venekamp et al. 2022 | General population | Asymptomatic | CLINITEST (Siemens Healthineers) | RT-PCR | 1344 | 25.63 | 99.92 |
