# Supplementary material for: Self-tests for COVID-19: What is the evidence? A living systematic review and meta-analysis (2020–2023)
Source: PLOS Glob Public Health. 2024 Feb 7;4(2):e0002336. doi: 10.1371/journal.pgph.0002336 (PMC10849237; doi:10.1371/journal.pgph.0002336)
Supplement: S4 Table — A-C Risk of bias. (DOCX) [file pgph.0002336.s005.docx]

**S4 Table A Risk of bias assessment results: QUADAS-2 (total studies=14). Low, high, and unclear chance of bias are represented by a green, red, and blue symbol, respectively.**

| Study ID | Author Name, Year | RISK OF BIAS | | | | APPLICABILITY CONCERNS | | |
| --- | --- | --- | --- | --- | --- | --- | --- | --- |
|  |  | **PATIENT SELECTION** | **INDEX TEST** | **REFERENCE STANDARD** | **FLOW AND TIMING** | **PATIENT SELECTION** | **INDEX TEST** | **REFERENCE STANDARD** |
| 1 | Harmon et al., 2021 | ☺ | ☺ | ? | ☺ | ☺ | ☺ | ☺ |
| 2 | Lindner et al., 2021 | ☺ | ☺ | ☺ | ☺ | ☺ | ☺ | ☺ |
| 11 | Schuit et al., 2021 | ☺ | ☺ | ☺ | ☺ | ☺ | ☺ | ☺ |
| 14 | Moller et al., 2022 | ? | ? | ? | ☺ | ☺ | ☺ | ☺ |
| 16 | Frediani et al., 2021 | ☹ | ☹ | ? | ☺ | ☺ | ☺ | ☺ |
| 17 | Stohr et al., 2021 | ☺ | ☺ | ? | ☺ | ☺ | ☺ | ☺ |
| 26 | Kim et al., 2021 | ☹ | ☹ | ☺ | ☺ | ☹ | ☺ | ☺ |
| 27 | Tonen-Wolvec et al., 2021 | ☺ | ☺ | ? | ☺ | ☺ | ☺ | ☺ |
| 30 | Zwart et al., 2022 | ☺ | ☺ | ☺ | ☺ | ☺ | ☺ | ☺ |
| 34 | Peto et al., 2021 | ☺ | ☺ | ? | ☺ | ☺ | ☺ | ☺ |
| 39 | Garcia-Finana et al., 2021 | ☺ | ☺ | ☺ | ☺ | ☺ | ☺ | ☺ |
| 49 | Schuit et al., 2022 | ☺ | ☺ | ☺ | ☹ | ☺ | ☺ | ☺ |
| 54 | Leventopoulous et al., 2022 | ☺ | ☺ | ☺ | ☺ | ? | ☺ | ☺ |
| 69 | Venekamp et al., 2022 | ☺ | ☺ | ☺ | ☺ | ☺ | ☺ | ☺ |

**S4 Table B Risk of bias for cohort studies: Newcastle-Ottawa scale (total studies=13). Stars represent the score according to the NOS scale by category**

| **Study ID** | **Author Name, Year** | **Selection (maximum 4)** | **Comparability (maximum 2)** | **Outcome (maximum 2)** |
| --- | --- | --- | --- | --- |
| 9 | Hughes et al., 2022 | *** | 0 | ** |
| 12 | Lamb et al., 2021 | *** | ** | * |
| 15 | Hirst et al., 2021 | *** | ** | * |
| 18 | Wachinger et al., 2021 | ** | 0 | ** |
| 19 | Wanat et al., 2021 | *** | * | ** |
| 22 | Kheirodin et al., 2021 | **** | * | ** |
| 24 | Denford et al., 2021 | **** | * | ** |
| 42 | Soni et al., 2022 | **** | * | **** |
| 44 | Herbert et al., 2022 | **** | 0 | *** |
| 45 | Herbert et al., 2022 | **** | * | * |
| 46 | Herbert et al., 2022 | ** | * | ** |
| 56 | Coller et al., 2022 | *** | * | * |
| 65 | O’Byrne et al., 2022 | **** | ** | 0 |

**S4 Table C Risk of bias for cross-sectional studies: Newcastle-Ottawa scale (Total studies=41). Stars represent the score according to the NOS scale by category**

| **Study ID** | **Author Name, Year** | **Selection (maximum 5)** | **Comparability (maximum 2)** | **Outcome (maximum 3)** |
| --- | --- | --- | --- | --- |
| 3 | Mistler et al., 2022 | ** | ** | ** |
| 4 | Betsch et al., 2021 | *** | ** | ** |
| 5 | Bien-Gund et al., 2021 | **** | ** | ** |
| 6 | Bouillat et al., 2021 | **** | * | * |
| 7 | Thomas et al., 2022 | *** | 0 | ** |
| 8 | Thomas et al., 2022 | ***** | ** | ** |
| 10 | Mouliou et al., 2021 | *** | ** | * |
| 13 | Philips et al., 2021 | *** | ** | ** |
| 20 | Cassuto et al., 2021 | ** | 0 | * |
| 21 | Goggolidou et al., 2021 | ** | ** | ** |
| 23 | Willeit et al., 2021 | *** | * | ** |
| 25 | Hoehl et al., 2021 | ** | 0 | ** |
| 29 | Prazuck et al., 2021 | ** | 0 | * |
| 31 | Undelikwo et al., 2022 | * | ** | ** |
| 32 | Love et al., 2021 | **** | ** | *** |
| 33 | Martin et al., 2021 | *** | ** | ** |
| 35 | Downs et al., 2021 | *** | 0 | *** |
| 36 | Tulloch et al., 2021 | **** | 0 | ** |
| 37 | Institute of Population Health, 2021 | **** | ** | ** |
| 38 | Sibanda et al., 2022 | *** | * | ** |
| 40 | Shilton et al., 2022 | *** | * | * |
| 41 | Jairoun et al., 2022 | ** | ** | ** |
| 43 | Rader et al., 2022 | *** | 0 | ** |
| 47 | LeRouge et al., 2022 | *** | * | ** |
| 48 | D’Agostino et al., 2022 | *** | ** | ** |
| 50 | Marinos et al., 2022 | *** | * | ** |
| 51 | Martinez-Perez et al., 2022 | **** | * | ** |
| 52 | Papenburg et al., 2022 | ***** | * | ** |
| 53 | Ritchey et al., 2022 | **** | 0 | * |
| 55 | Coker et al., 2022 | *** | ** | ** |
| 57 | Qasmieh et al., 2022 | *** | * | ** |
| 58 | Nwaozuru et al., 2022 | ** | 0 | ** |
| 59 | Agusti et al., 2022 | **** | ** | ** |
| 60 | Bae et al., 2022 | *** | * | ** |
| 61 | Dallera et al., 2022 | *** | 0 | ** |
| 62 | Daniore et al., 2022 | **** | * | ** |
| 63 | Fishman et al., 2022 | ** | ** | ** |
| 64 | Hajek et al., 2023 | *** | 0 | ** |
| 66 | Qasmieh et al., 2022 | **** | ** | ** |
| 68 | Stemler et al., 2022 | *** | * | ** |
| 70 | Wu et al., 2023 | **** | ** | * |
