# Supplementary material for: Self-tests for COVID-19: What is the evidence? A living systematic review and meta-analysis (2020–2023)
Source: PLOS Glob Public Health. 2024 Feb 7;4(2):e0002336. doi: 10.1371/journal.pgph.0002336 (PMC10849237; doi:10.1371/journal.pgph.0002336)
Supplement: S1 Fig — A-D Comparison of summary receiver operating characteristic (SROC) curves. (DOCX) [file pgph.0002336.s006.docx]

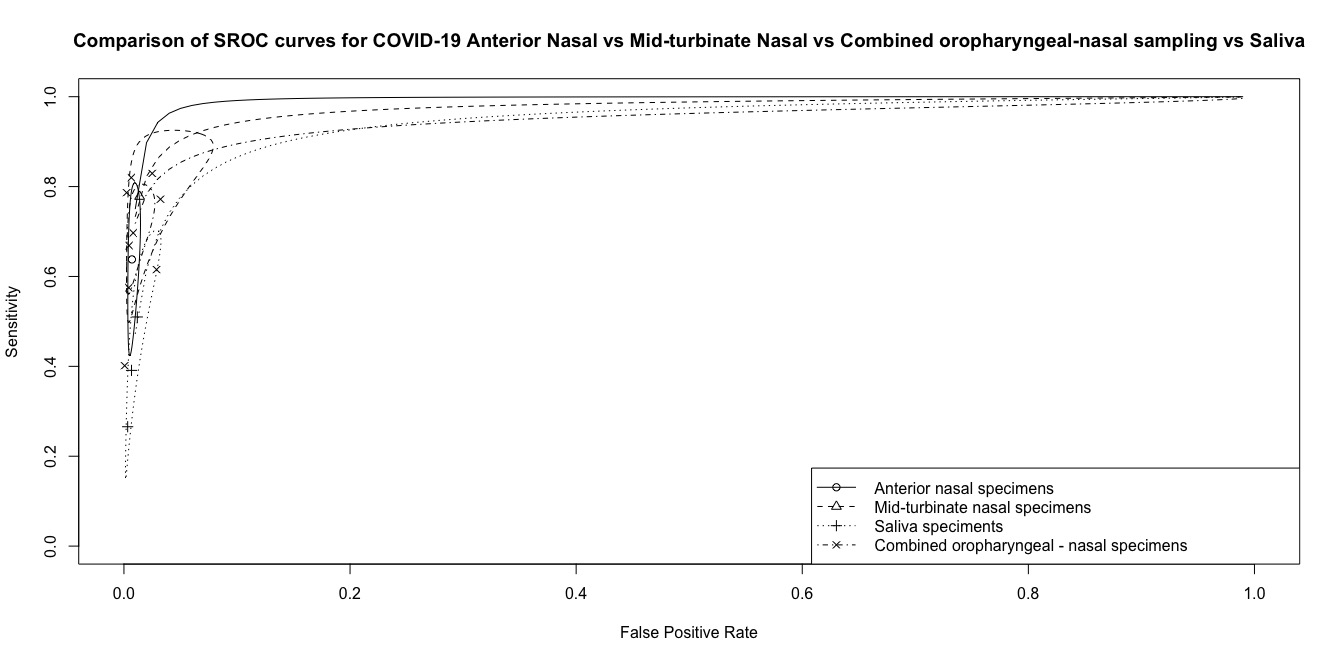


**S1 Fig A Comparison of summary receiver operating characteristic (SROC) curves for various self-sampling sites (anterior nasal, mid-turbinate, combined oropharyngeal-nasal, and saliva) for COVID- 19 self-testing**


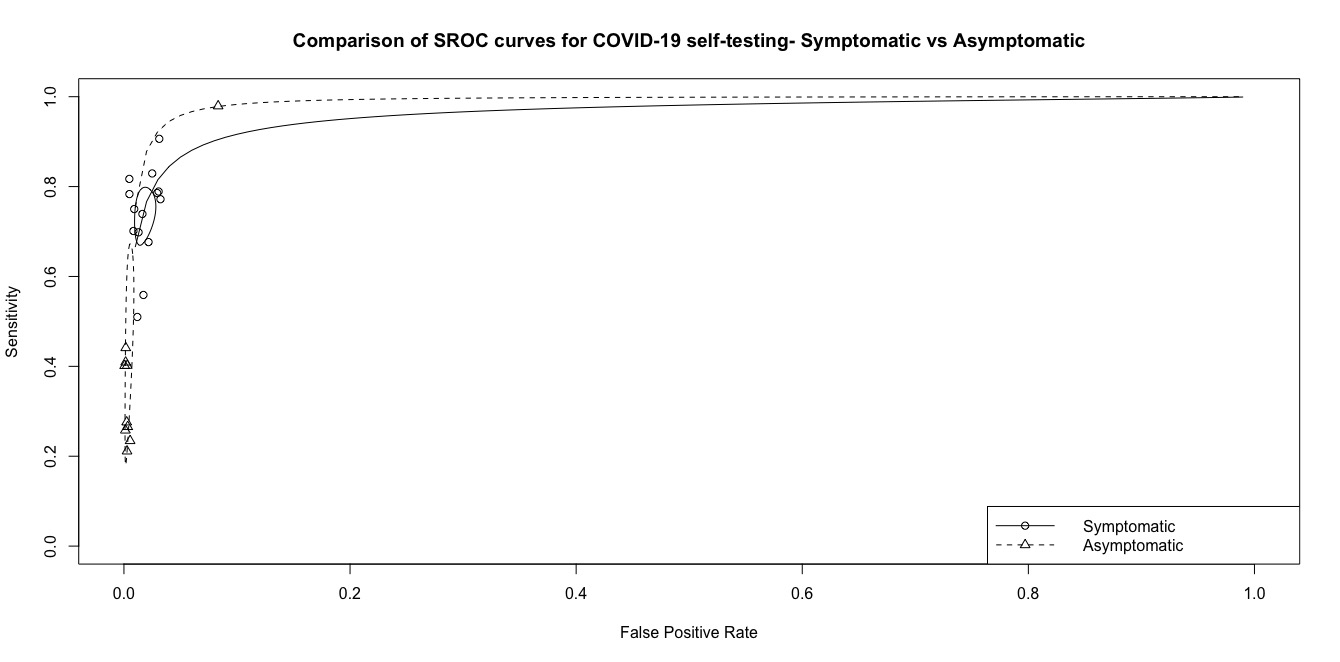


**S1 Fig B Comparison of summary receiver operating characteristic (SROC) curves based on symptomatic status (symptomatic versus asymptomatic) for COVID-19 self-testing**


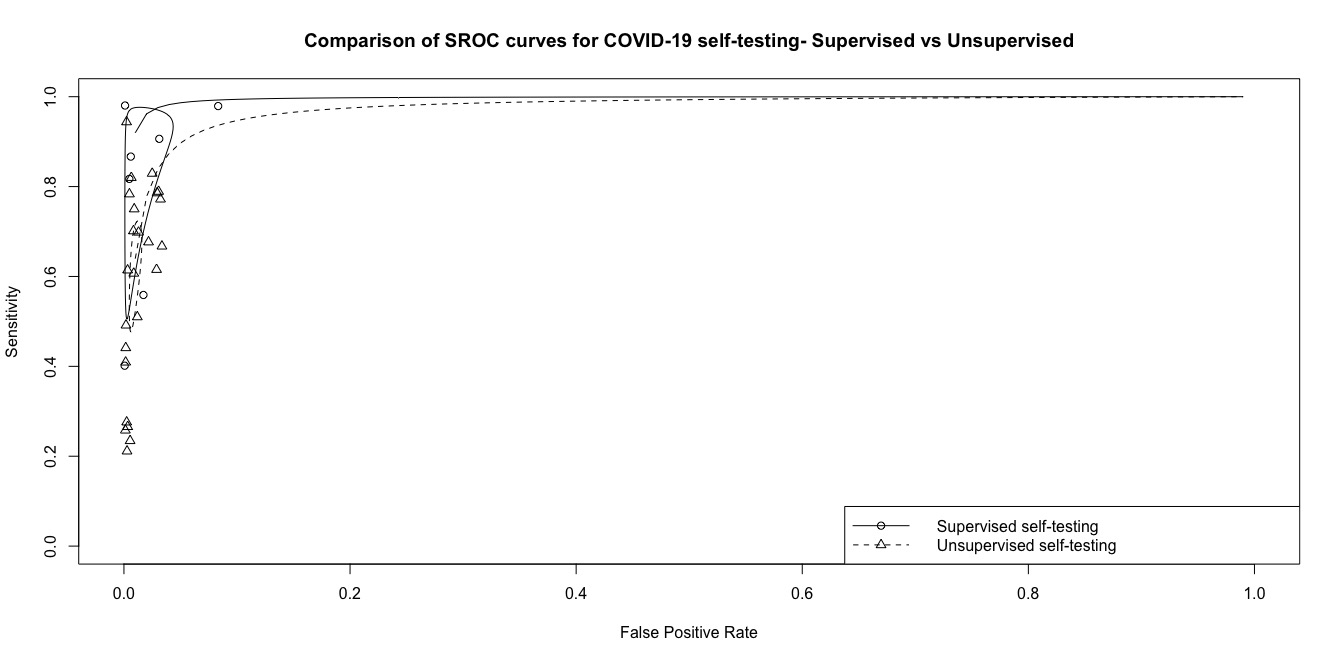


**S1 Fig C Comparison of summary receiver operating characteristic (SROC) curves based on testing method (supervised versus unsupervised) for COVID-19 self-testing**


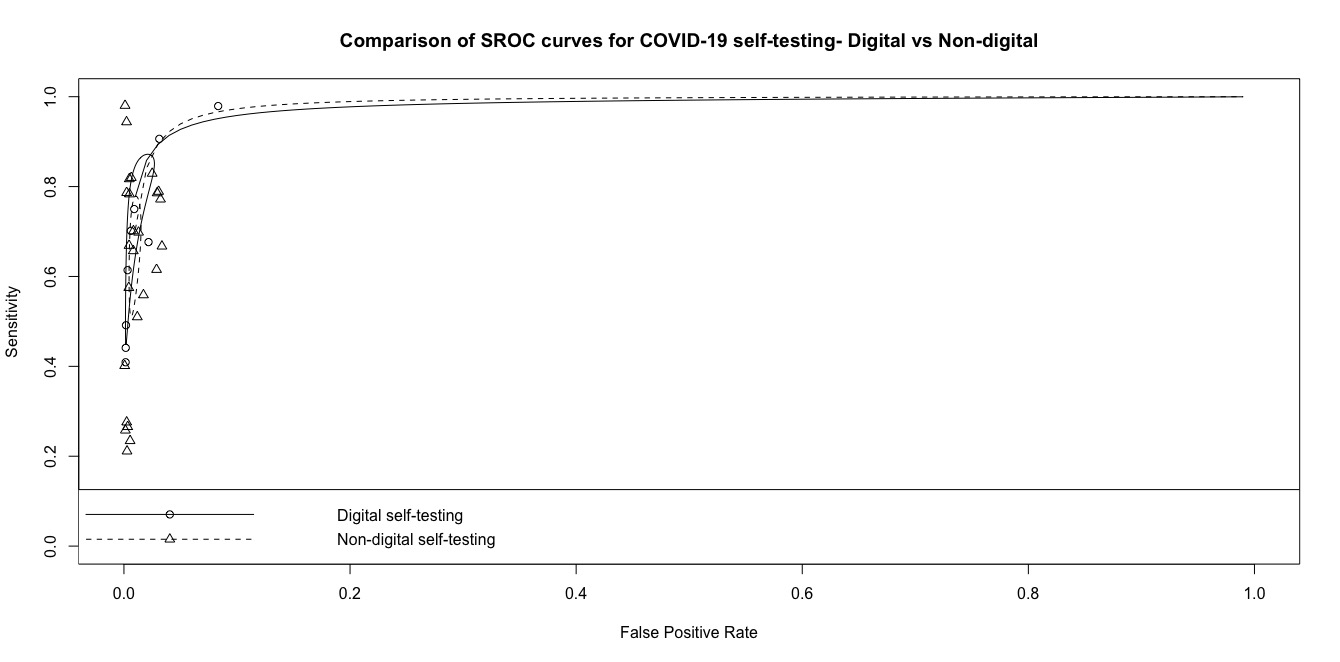


**S1 Fig D Comparison of summary receiver operating characteristic (SROC) curves based on the presence of a digital intervention for COVID-19 self-testing**
