# Supplementary figures and images for: Self-tests for COVID-19: What is the evidence? A living systematic review and meta-analysis (2020–2023)
Source: PLOS Glob Public Health. 2024 Feb 7;4(2):e0002336. doi: 10.1371/journal.pgph.0002336 (PMC10849237; doi:10.1371/journal.pgph.0002336)

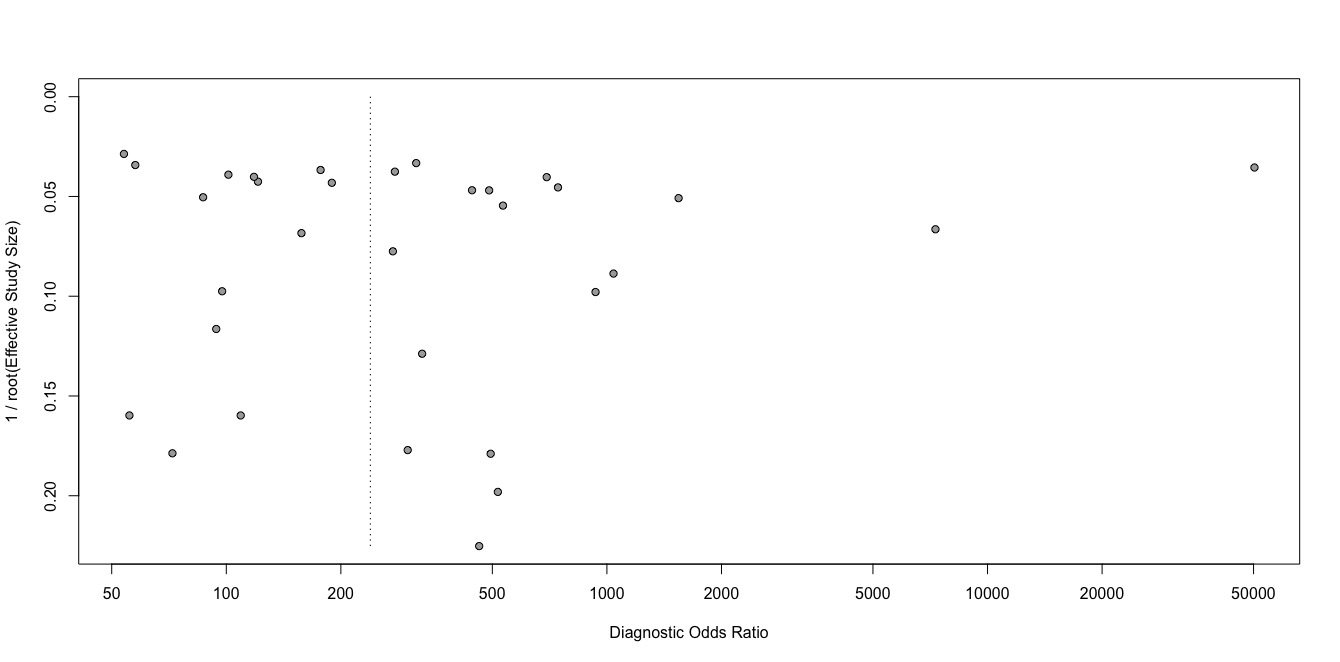


**S2 Fig Funnel Plot of studies included in the meta-analysis (n=14)**

Supplement: S2 Fig — (DOCX) [file pgph.0002336.s007.docx]
